# Innovative approaches to HIV/AIDS financing: lessons learned from the Sustainable Financing Initiative (SFI)

**DOI:** 10.1186/s12913-025-12529-8

**Published:** 2025-05-28

**Authors:** Susanna Baker, Mai Hijazi, A. K. Nandakumar

**Affiliations:** 1 Office of HIV/AIDS, Global Health Bureau, U.S. Agency for International Development, Washington D.C., USA; 2 Office of Health Systems, Global Health Bureau, U.S. Agency for International Development, Washington D.C., USA; 3 The Heller School of Social Policy and Management, Brandeis University, Waltham, MA USA

**Keywords:** HIV/AIDS, HIV/AIDS financing, Financial protection, Health financing, Health systems strengthening, Sustainability

## Abstract

**Introduction:**

As of 2018, domestic resources covered 56% of the total financing for the HIV/AIDS response in low- and middle-income countries (UNAIDS, AIDSInfo - Global data on HIV epidemiology and response, 2021). This has not been sufficient to close the financing gap as donor funding, such as PEPFAR, flatlines or declines in many countries. The Sustainable Financing Initiative (SFI) was a PEPFAR-funded, USAID-led initiative aimed at leveraging the rapid economic growth in many focus countries to increase domestic funding for HIV/AIDS.

**Methods:**

SFI worked with ministries of health and finance, the private sector, and other critical stakeholders to implement innovative health financing solutions aimed at increasing domestic spending on HIV/AIDS. Three core areas were emphasized in SFI’s approach: improved public financial management, integration of HIV services into social health insurance schemes, and greater private sector engagement in the financing and delivery of HIV services. SFI supported these areas through advocacy, evidence generation, strategic engagement with country governments, the use of metrics to measure results, willingness to take measured risks, and the readiness to stop funding activities that did not yield results. Through new partnerships and ways of doing business, SFI worked to increase the efficiency and sustainability of the HIV response. A core principle underlying SFI was to make system changes that would lead to sustained long-term increases in domestic spending on HIV/AIDS.

**Results:**

SFI invested PEPFAR funding across sixteen countries and two regional programs to leverage domestic resources for HIV/AIDS and improve approaches to health financing. With an investment of $47.8 million, SFI generated an estimated $393 million in domestic resources for HIV. Additionally, SFI interventions yielded significant results globally, generated lessons learned and made strides towards improving sustainability of HIV programming.

**Conclusion:**

By implementing innovative approaches to HIV financing and how interventions are designed and implemented, PEPFAR funding can leverage additional resources from host-country governments and the private sector for a more sustainable HIV response.

## Background

The Sustainable Financing Initiative (SFI) for HIV/AIDS was a PEPFAR funded, USAID-led initiative which was started in 2014 as a bold vision for increasing domestic resources for HIV/AIDS. With a budget of $47.8 million, SFI supported 16 countries and two regional programs to further mobilize and more effectively use their own resources to address the needs of people living with HIV/AIDS.

As of 2018, domestic resources covered 56% of the total financing for the HIV/AIDS response in low- and middle-income countries [1]. This has not been sufficient to close the financing gap as donor funding, such as PEPFAR, flatlines or declines in many countries. The original concept for SFI was based on the idea that many PEPFAR countries have experienced significant economic growth in recent years, which implies greater country capacity and willingness to spend on their own health needs. The initiative sought to assist countries to tap into this economic potential and facilitate increased capacity to mobilize health resources in an efficient and effective manner to target those in need. SFI partnered closely with select USAID country offices (known as missions) and relevant stakeholders to support new and ongoing health financing activities to increase domestic resources for the health sector.

## Program approach

As a World Bank report stated, “Although development assistance can have an important catalytic function for countries to finance, domestic resources for health will need to increase in the longer term in order to ensure more predictable and sustainable funding” [2]. With this underlying assumption, SFI worked with ministries of health and finance, the private sector, and other critical stakeholders to implement focused and strategic health financing solutions to increase domestic resources available for HIV/AIDS.

Three core areas were emphasized in SFI’s approach: improved public financial management, integration of HIV services into financial protection schemes, and greater private sector engagement. SFI supported these areas through advocacy and evidence generation. It is important to note SFI did not focus on tax reform or innovative tax schemes. Tax reform is a critical piece to raising new money for health [2, 3]. However, it was felt to be beyond the manageable scope of SFI, and SFI instead focused on increasing efficiency and advocacy for domestic resources available for HIV.

### Public financial management (PFM)

Public financial management (PFM) is the process by which governments manage public resources, including revenue and expenditure. Important key PFM functions include budgeting and resource mobilization, which are two areas on which SFI focused. Improved PFM systems, through improved spending efficiency or stewardship of public funds, leads to greater collection of domestic resources as citizens see the clear impact of their tax contributions. Runde and Savoy note that the increased focus on domestic resource mobilization as a stable source of long-term funding is welcome, but note that, “Simply improving the ability of a country to raise additional tax and revenue from its citizens without ensuring that these resources will be well spent will not achieve the desired development outcomes. This will require a much greater focus on public financial management (PFM)” [4]. SFI interventions focused on PFM to help mobilize country resources for the health and HIV response and maximize the impact of existing investments by providing technical assistance and training on budget al.location and execution to government officials and other stakeholders at the national, sub-national, and local levels. PFM work included both capacity building for management of funds as well as evidence generation and advocacy to increase domestic resources for HIV/AIDS. These two PFM functions relate to the goal of resource mobilization but in different ways. Capacity building around the management of funds has the potential to increase efficiencies and gains in resource utilization, while advocacy for increased resources has the potential to lead to more resources being available overall.

### Financial protection

Financial protection work focused on analysis and advocacy to add HIV services into existing health insurance plans, enroll more PLHIV into health insurance, contract HIV providers with plans, improve the efficiency of HIV commodity procurement, and increase domestic funding for HIV through the insurance plans. The work also supported integrating HIV into broader universal health coverage (UHC) efforts and worked to ensure PLHIV were not charged user fees for services. It is important to note that while insurance provides a vehicle for mobilizing additional funds, its presence is limited in the countries in which PEPFAR works and in many cases, resources are required to design and provide evidence before these mechanisms are able to mobilize funds. Increasing financial risk protection in ways other than health insurance, such as elimination of fees, is a positive benefit, but requires additional resources and doesn’t in and of itself mobilize them. Ooms and Kruja emphasize that all national health insurance schemes need to have mechanisms to avoid the exclusion of people who are unable to contribute, and they highlight the risks associated with integration of HIV into national health insurance schemes (NHI). They note NHI schemes require regular contributions to function and that not all PLHIV might be able to contribute [5]. 

### Private sector

Evidence on the impact of involving the private sector in the provision of HIV care is still growing, however the existing literature highlights the potential for governments to scale up healthcare financing by leveraging private resources, innovations, and expertise [6]. Under SFI, private sector work helped more PLHIV get the care they need. Service delivery interventions included enabling the provision of publicly financed care from private providers. Private providers were used to distribute donor or government-funded commodities and services. Market-based approaches were also implemented, in which clients voluntarily bought HIV products and services in commercial settings. Financing interventions sought to mobilize resources from private companies, banks, and households. Strategies included blended finance approaches which used U.S. government-backed loan guarantees, capacity building to incentivize greater private lending to the health sector and capacity building to NGOs to launch revenue-generating social enterprises. Engagement with the private sector requires resources but has the potential to increase resources through co-financing with private sector funds or efficiencies. Additionally engaging the private sector allows for patient choice and in some cases increased clinical outcomes.

Key principles were established at the beginning of SFI which guided the decisions on where and how to invest. These included rigorous metrics and benchmarks; a willingness to halt investments that didn’t yield results; potential impact in a defined timeframe; a demonstrated return on investment; increased service coverage for prevention, care, and treatment for HIV/AIDS; the need to tailor interventions for individual country contexts; and coordination and collaboration across other Agencies (e.g. US Treasury) and with multilateral stakeholders (e.g. The Global Fund, World Bank and WHO).

Systems strengthening work is challenging to measure and SFI aimed to show that this type of work could yield concrete results in a 3–5-year time frame. The idea of a demonstrated return on investment (ROI) was core to SFI, although in some cases there was no ROI, or a negative ROI and the investment was still seen as important or worthwhile. For example, some policy work that was critical for laying the foundation for future financing work was not quantifiable with an ROI but was still seen as foundational to achieving financing results and could yield positive ROI in future years.

Since SFI was PEPFAR funded, all the investments needed to be designed to positively impact the HIV/AIDS response. While in several cases, SFI investments had positive spillover effects for overall health financing in the country, HIV/AIDS was always the starting point for designing interventions and metrics had to show how the intervention would positively impact the HIV/AIDS portfolio.

When deciding where to invest, several criteria were used to select countries. These criteria included political commitment and demonstrated host country interest in increasing domestic resources for HIV/AIDS, prioritization of health programs in country, macroeconomic growth potential, potential for private sector development (e.g. positive regulatory environment for the private sector, positive investor outlook or interest in private sector health service delivery, large numbers of patients already seeking care in the private sector), in country commitments to explore efficient approaches to service delivery, USAID country office capacity and interest in managing a sustainable financing portfolio, and anticipated future trends in PEPFAR and/or Global Fund support. These criteria were intended to choose countries to invest where the enabling environment and mission interest and capacity were such that the financing interventions had the greatest chance for positive impact.

## Methodology

Calculating ROI for a diverse set of activities, countries, and timeframe proved challenging. Data availability and the nature of the activities made it impossible to have a truly uniform approach to calculating ROI across activities. However, a deliberate attempt was undertaken to calculate as accurate a ROI as possible and that methodology will be discussed here. ROI is defined as the net financial benefit of an intervention divided by the initial investment (expenditure) to implement that intervention or (return - expenditure)/expenditure.

Methodology differed according to the pillar. For PFM, the ROI was calculated as the total incremental funds executed and/or allocated because of the activity. For financial protection, ROI was calculated as the government or client contribution for insurance payments, including premiums, user fee subsidies and reimbursements. In some cases, this is from government budget data and in other cases this is estimated using the payment rates multiplied by the number of beneficiaries. Under the private sector engagement pillar there was private service delivery and private financing. For private service delivery, ROI was calculated as the total number of services delivered in the private sector (test kits, ARV refills, or viral load tests) multiplied by the estimated variable unit cost of service delivery in the public sector. The variable unit cost was estimated using published literature from each country. This calculation estimates the cost savings to the government of shifting patients out of the public sector. For private financing, ROI was calculated as the total funds contributed from private organizations or clients. When activities were cross-cutting across the three pillars, ROI was only captured in one pillar to avoid double counting.

There are limitations that are important to note. Firstly, it is difficult to directly attribute financial returns to the SFI investment. These efforts by SFI should be seen as a significant contribution to the results, rather than a direct and exclusive attribution. Secondly, figures were not adjusted to present dollars for simplicity. Ideally, a net present value should be used to adjust expenditures and return to present dollars, especially when looking at long time horizons. For sake of simplicity, these analyses did not calculate a net present value (NPV). Also, some returns reflect benefits to the broader health system, not just for HIV, while some activities were more narrowly focused on HIV. Lastly, the time horizon for benefits in these ROI calculations are only during the period of implementation. ROI is expected to grow as results from interventions continue into the future and as allocated budgets are spent. This figure does not include budget allocations or projected ROI where policies are pending implementation.

Activities could be categorized into four main categories for ROI. The first category was where the activity clearly produced a measurable positive ROI and included many of the PFM activities and the private financing activities. The second category included activities where there was a negative ROI. These activities included some of the private service delivery activities, which in many cases required large upfront investments. It is hoped as the benefits of the activity continue to accrue over time, the ROIs will become positive. The third category included activities where it was not possible to estimate an ROI due to the nature of the work and the time frame required to see impact. This included research and evidence generation, policy and advocacy work, or capacity building efforts. If policies are adopted and reform undertaken, the ROI from this work could be positive over the long-term. Fourth and finally, the last category is where activities were halted or not fully started due to lack of progress or change in priorities within the governments and therefore no ROI was calculated. This category includes the National AIDS Trust Fund work in Tanzania, community-based health insurance program in Rwanda, and PFM work in Rivers State in Nigeria, where SFI work was halted.

## Results

With an investment of $47.8 million, SFI contributed to generating at a minimum $393 million in domestic resources for health and HIV across the three pillars of public financial management, financial protection, and private sector engagement. A detailed breakdown of country specific results can be found in Table 1, while this section will cite some of the more significant results in each pillar and note which countries had the largest returns on investments.

**Table 1:** Summary of activities and results

Country	Activities Implemented	Amounts Invested	Estimated ROI	Discussion/comments
Botswana	PFM - Harmonizing separate System of Health Accounts (SHA) and National AIDS Spending Assessment (NASA) tools and institutionalizing health and HIV expenditure tracking - Developed and rolled out Financial Management training at the sub-national and national levels - Advocated for Government of Botswana (GOB) to adopt pooled procurement using evidence FP - Collaborated with the GOB and Medical Aid Schemes to improve cost recovery for the HIV response - GOB with cost analyses of an HIV Basic Service Package as part of the Universal Health Services Package (UHSP) insurance solution PS - Trained senior hospital staff on contracting and public-private partnerships. - Organized workshops including private sector partners and government partners to pursue public-private partnerships.	$2.1 M	N/A	No concrete ROI due to the activities being higher level policy work that is harder to quantify in the short term or to contextual issues. At end of SFI, it was too early to tell how the governments will choose to move forward with the analysis that was produced.
Cambodia	FP - Assistance to the National AIDS Authority (NAA) to develop a national strategy for HIV-related sustainability and raise domestic funding - Evidence generation for budget allocation for the integration of HIV activities and the use of health facility funds for HIV services - Costing and financing the National Strategic Plan for the Royal Government of Cambodia (RGC) HIV response - Technical support in HIV-related beneficiaries’ coverage by and use of Health Equity Fund (HEF) and other health insurance schemes - Private sector engagement assessments to explore contributions to HIV/AIDS prevention and treatment efforts and financing	$1,204,985	$16,140,000	The Government of Cambodia adopted a policy circular which allowed for many of the reforms needed and led to the ROI. Private sector activities were halted. SFI work focused on incorporating HIV into existing health protection schemes–resulting in 100% of PLHIV eligible to access free healthcare services in Cambodia, up from only 38% in 2018
Cameroon	FP - Developed a communication strategy to inform the public and health service providers about the elimination of HIV user fees - Supported the MOH in updating its strategic communication plan for UHC. PSE - Decentralized delivery of ARVs through the private sector by evaluating the feasibility of several decentralized models, including private pharmacies, clinics, faith-based organizations, community pickup points and home delivery	$1 M	N/A	The FP activities were fundamental for reforms which are to come and are expected to contribute to ROI. PSE activities have just now (2023) begun enrolling patients and will also contribute to an ROI
Côte d’Ivoire	PFM - Strengthening the capacity of the public sector to provide oversight and leadership of HIV programs at central and decentralized levels PSE - Ensure active private sector participation in the HIV Task Force of the National Coordination Platform for Health Financing - Assessing the feasibility of a model for decentralized antiretroviral (ARV) drug distribution at the community level FP - Analysis on PLHIV user fee elimination to ensure no user fees charged	$1 M	N/A	SFI activities were mostly focused on governance activities which take longer to contribute to ROI. SFI supported a study on user fees to assess whether user fees were being charged to PLHIV, but the study had not yet led to any concrete action
Dominican Republic	PFM - Generation of evidence and advocacy efforts to ensure funding for the HIV response, with particular focus on funding ARVs through the Government of the Dominican Republic (GODR) annual budget process. PSE/FP - Supported non-governmental organizations’ participation in the national health insurance scheme - Advocated for the inclusion of ARVs in insurance benefit packages through advocacy and community outreach to clients and health clinics. - Strengthened the capacity of health workers to increase health insurance enrollment for PLHIV.	$570,000	$ 434,100	Work to include ARVs into insurance benefit packages will continue to contribute to ROI and this ROI figure is likely much higher a few years out. SFI support for advocacy and awareness raising resulted in a 38% increase of PLHIV enrolled in the National Health Insurance Program. SFI supported four NGOs with social enterprise models that included business planning and capacity building for revenue generation. As a result, one of these organizations is in the process of designing a new dermatology wing that will leverage insurance payments for curative care and out-of-pocket payments for cosmetic services to cross-subsidize HIV care for vulnerable populations. ROI is not yet available for this activity.
Ethiopia	PFM - SFI collaborated with the Federal HIV/AIDS Prevention and Control Office to develop a DRM strategy that aims to increase domestic financing - Reviewed the existing tax structure in Ethiopia to identify possibilities to improve HIV funding - Identified innovative financing and other health sector entities that could play a larger role in helping fund the domestic HIV response PSE - Provided evidence needed to expand access to and use of HIV services in the private sector, identifying any legal or regulatory barriers for the private sector to provide HIV services - Supporting analysis of client preferences, willingness to pay, and providers’ preferences	$150,000	N/A	Activities focused on evidence generation needed for advocacy. However, conflict in Ethiopia halted additional activities
India	PFM/PSE - A formal market assessment of HIV case finding and linkage to antiretroviral treatment (ART) treatment services in Thane and Pune. - Design a strategic purchasing model and support framework, including mechanisms to monitor outcomes and a proposed implementation plan with inputs from key stakeholders - Preliminary testing of an outcome-based strategic purchasing model for HIV case finding, treatment initiation, and treatment continuation in Thane and Pune.	$500,000	N/A	Work is ongoing and results are still to come. The Government of India (GOI) recently approved the model to move forward
Kenya	PFM - Advocacy for increased line item funding to HIV in national budgets, county-level PBB capacity building, evidence generation for resource mobilization	$4,311,465	$257,518,439	This PFM technical assistance was timely and aligned with governmental budget reforms will allow success and a large ROI. Advocacy work restored government funds for ARVs, with the commitment growing from US$20 million to US$39 million–however, the execution of this budget line item remains inconsistent.
Namibia	PSE - Conduct a market assessment and willingness to pay study for PrEP in the private sector similar to previous work done for HIVST Kits. - Analysis of provider payment modalities PFM - Supporting sustainable financing, efficiency improving, and capacity for private sector contracting in Namibia along with supporting regional learning between Namibia, South Africa, and Botswana	$2.2 M	N/A	SFI activities were mostly focused on policy activities which take longer to contribute to ROI.
Nepal	PFM - Support to government on cost/financial gap estimates for next Nepal Health Sector Support Programme (NHSP) - Support to sub-national governments on appropriate planning and budgeting tools to allocate funds to HIV	$500,000	N/A	SFI activities were mostly focused on policy activities which take longer to contribute to ROI.
Nigeria	PSE - Market analyses and strengthened the capacity of the private clinics and hospitals to expand HIV service delivery - Fostered innovative partnerships with private sector laboratories and lab equipment manufacturers to improve the procurement and distribution of viral load testing for PLHIV in Lagos and Rivers States - Decentralized drug distribution model with private pharmacists and clinics to expand access to care in the private sector PFM - Provided technical assistance to help develop the HIV Domestic Resource Mobilization Strategy, - Worked with government officials in Kano and Lagos states to improve their budget planning FP - Supported analysis, advocacy, and engagement with authorities in Lagos and Kano states to help develop a roadmap for integrating HIV services into the state’s social health insurance scheme.	$ 2,607,744	$4,419,335	SFI work focused on ensuring that HIV was included as the financial protection schemes were created. As a result, in Lagos state this led to the enrollment of more than 541,000 people into the health insurance scheme SFI helped two private labs lease equipment so that the lab could sell viral load testing services to clients at market prices and negotiated with self-test kits manufacturers to sell kits at reduced prices to private pharmacies where clients could purchase them. Uptake of the viral load testing and HIVST activities in Nigeria were lower than anticipated, largely due to the availability of free or lower priced alternatives
Rwanda	FP - Costing inclusion of HIV, TB and Hep into Community Based Health Insurance (CBHI)	$100,000	N/A	Activity was halted before work could begin. There was initial interest for work on the community-based health insurance program–however the work stalled due to delays and the funding was moved elsewhere.
Tanzania	PFM - Health budget Analysis, HIV Service Delivery Efficiency Analysis, and Assessment of GOT’s Budget Execution Performance on Allocations to HIV PSE - Developed partnership with nonprofit Trafigura-North Star to expand availability of HIV services - Training of private sector nurses to expand HIV services at nurse-led private clinics - coached 70 facilities on loan application processes and helped develop business cases to incentivize lending FP - Built the capacity of private insurance companies to increase affordability and availability of HIV services and support enrollment of PLHIV in private health insurance	$3,595,000	$68,881,814	SFI supported a pilot that allowed 200 clinically stable clients who were willing and able to pay a small delivery fee of around $4 to have their ARVs delivered to the community. However, the pilot was put on hold due to challenges with fee collection and requests from clients for additional services that couldn’t be provided. PFM work included a multi-million dollar budget allocations for essential health commodities, including ARVs in 2016 and 2017.
Uganda	PFM - Improve budget planning and spending at the local and national levels, by supporting the MOH transition to program-based budgeting (PBB), - Support for a health budget execution bottleneck analysis that informed interventions to address inefficiencies - Revision of tax management procedures increased Uganda Revenue Authority (URA) revenue collection - Investment to improve coordination in procurement, contract management, and payments in the Ministry of Health	$ 1,869,000	$ 17,429,727	Budget execution rates at MoH increased from 80% in 2015/16 to 97% in 2017/18, leading to an additional US$17.4 million in health spending.
Vietnam	PFM - Training and technical assistance to strengthen the Government of Vietnam’s HIV budget and expenditure analysis, with a focus on ARV procurement and management - Support local governments customize their copayment subsidy models and update their cost estimates for copayment subsidy needs. FP - Support to strengthen policy and legal frameworks to increase the number of PLHIV enrolled in SHI, ensure financial protection for PLHIV through provincial government subsidies for premiums and copayments, and integrate stand-alone HIV facilities into the country’s public health system to meet SHI reimbursement eligibility requirements	$ 4,312,857	$ 26,394,622	SFI work focused on incorporating HIV into existing health protection schemes–resulting in 90% of PLHIV enrolled in SHI
Zambia	PSE - Evidence generation, advocacy, coordination and implementation plan for competitive pricing of ARVs in the private sector - Support for decentralized drug pick up	$ 700,000	$ 1,653,143	16,665 clients enrolled in the decentralized drug pick up or central dispensing unit (CDU) program, representing over half of those eligible in participating health facilities, and 97% of CDU clients were retained after 36 months.
Asia Regional Program	PSE - Analysis for market development for HIVST, condoms and ARVs	$266,666	N/A	Only analysis was completed
Caribbean Regional Program	PSE - Supports establishment of a fund in the Caribbean that will serve as a source of financing for donor-dependent CSOs and NGOs that support KPs in the region	$ 400,000	$ 137,000	
Total		$27,937,717	$393,008,180	

*N/A* The reason why the ROI was not able to be calculated is explained in the Discussion/comments section for each country

### Public financial management (PFM)

SFI work contributed to significant increases in budget allocation and spending in five out of the nine countries that focused on building capacity to improve budgeting and execution of health and HIV/AIDS funds yielded significant results [7].

There was a positive return on investment (ROI) for many of the PFM activities in the SFI countries. Kenya had the largest estimated ROI, at more than 50:1 (for every dollar invested, an additional 50 dollars was generated). This was followed by Nigeria (36:1), Tanzania (29:1), and Uganda (9:1). Ethiopia and Cambodia have a high potential of return on investment if strategies and policies are implemented, and commitments realized. For some activities it was not possible to estimate an ROI due to the nature of the policy work and the time frame required to see change between capacity building efforts and budget allocation and execution. PFM work consistently had strong results. This is partly because it is straightforward and relatively simple to measure results such as budget allocated and expended. Another possible reason is that this type of work is dependent on strong political will and commitment to PFM reforms and SFI countries were chosen based on their willingness to engage in these types of reforms. If a country was unwilling to engage in substantive reforms, they would not have been chosen for this type of work. Finally, for countries wanting to show strong commitment to owning their HIV response, budget allocation and execution is the clearest marker of this commitment. Governments frequently begin to increase their financial commitments by funding tangible items, such as commodities, and several governments added an ARV budget line item or recommitted funding for an ARV budget line item.

### Financial protection

SFI implemented financial protection activities in a total of ten countries and there were significant investments in five of these countries [8]. There were strong results in the financial protection pillar. For example, there was a ROI of 2.22 for every dollar invested in Vietnam. SFI work in Vietnam focused on incorporating HIV into existing health protection schemes–resulting in 90% of PLHIV enrolled in SHI in Vietnam. Many of the results were even more impressive when the fact that these types of interventions often require significant policy reforms over a multi-year timeframe. In countries with significant financial protection results, these results were due to consistent technical assistance and engagement over several years. Policy changes which led to results took time and there were some years without concrete results before the policy reforms took root and yielded results. Again, these reforms were predicated on strong government commitment and political will to enact the changes. Success was seen in countries or sub-national units of countries with this strong political will. Notably, success was only feasible where there was existing financial protection work underway to which SFI could contribute. SFI did not create any national or social health insurance schemes, which would have entailed large monetary contributions and would probably have had a negative ROI in the timeframe of SFI.

### Private sector engagement

SFI implemented private sector activities in 11 countries and two regional programs [9]. Service delivery interventions reached over 48,000 clients with testing, treatment, and viral load testing services across Zambia, Nigeria, and Tanzania. Importantly, clinical outcomes were as strong, or stronger than outcomes in the public sector across these three country programs. For example, 97% of clients obtaining ARV refills from private pharmacies in Nigeria were retained on care after 12 months, compared to 74% in the public sector. These clients also experienced shorter wait times of an average of 30 min compared to 2.3 h in the public sector.

The financial ROI from service delivery interventions was mixed. Minor or negative ROI was reported for some activities due to the large upfront investments, such as software, training, and infrastructure, needed to start these interventions and the short time horizon of the activities. However, Nigeria’s private pharmacy program generated a positive ROI of 1.45:1 over a three year period. As the costs of these services are spread out over additional years and more clients choose to use these services, the ROI and results will continue to grow. Importantly, in addition to their financial impact, many of these interventions had positive results in terms of patient experience, satisfaction, and clinical outcomes–such as retention of clients in care.

Although SFI only implemented a few private financing models, there was a large ROI and interest in the models tested. For example, in Tanzania, SFI trained a bank and private providers on borrowing and lending in the health sector, resulting in $6.4 million in loans issued to HIV facilities. The loans were used to purchase equipment by health providers to expand their HIV testing capacity, resulting in a 35% increase in HIV tests following the loans. This resulted in an ROI of $15.68 leveraged for every dollar invested, due to the relatively low implementation cost to USAID. These models take time and expertise to create and scale up, however there is potential for these models to leverage private financing and bring needed capital to the HIV response. These models should be given serious consideration as future sustainability plans are developed.

### Overall

While the overall ROI for SFI was positive (with a $47.8 million investment, $393 million was generated) this was largely due to high ROI on PFM activities in four countries: Kenya, Uganda, Tanzania, and Vietnam (see Table 1 for more details). These countries were in the process of undertaking major financing reforms and SFI was able to support these reforms with critical technical assistance at the right time. The governments had shown political commitment to undertaking financial reforms and were supportive of the work. In addition, SFI spent the most time and money in these countries. However, for at least 17 other activities, there was no calculable ROI. Some of the lack of ROI is due to relatively short time frames or focus on advocacy and evidence generation. For other countries, the activities were halted because of conflict, such as in Ethiopia, or lack of government commitment. Other activities had a negative ROI due to start up costs or the activity simply not providing the return expected, such as the Caribbean Regional Fund. Discussion should be had around what these results indicate for future funding of PFM, financial protection, and private sector work and whether a positive ROI is the most important indicator of success, whether efforts should be focused where there is only strong government commitment, and what a reasonable timeframe and budget is for consistent donor funding to see results.

This paper was drafted as SFI was concluding so the sustainability of the funding increases post-SFI intervention has not been tracked comprehensively. However, for the four countries with large positive ROI for PFM and financial protection work—Kenya, Uganda, Tanzania, and Vietnam—the reforms have been codified into law and into the system and therefore one would expect to continue to see the positive ROI returns for years to come. For other countries, such as Cameroon and Namibia, some of the policy work had just concluded at the end of SFI and one would expect to see an increase in ROI for those countries. For other countries, where the work was halted due to conflict (Ethiopia) or unfavorable conditions (Caribbean, some of the Tanzania work, and Rwanda) there is no post-SFI increase in ROI. A systematic review of the impact of SFI interventions in a few years would be a welcome addition to the literature.

## Lessons learned

SFI was an innovative approach to increasing domestic funding for HIV/AIDS in that the multi-year initiative allowed for trial and error of different approaches and provided clear lessons on how to approach this work in the future. SFI showed that a focus on system changes can yield long-term results and demonstrated the right conditions for these long-term results.

Firstly, and most importantly, SFI demonstrated that *dedicated HIV financing interventions can yield concrete results*. PEPFAR is a result driven initiative where data on the number of clients on treatment, tested, and reached with prevention services are easily accessible. This has been a large factor in the success of PEPFAR as the benefits of the USG investment in terms of lives saved are clear. Financing, sustainability, and systems strengthening work has generally been harder to quantify and justify investments. However, SFI showed that a focus on measuring financing work can lead to clear results in three to five years–both on clinical outcomes and systems outcomes–and that this work is a valuable use of funding.

However, the *conditions for investing in systems work must be favorable.* The overall positive ROI for SFI was mainly due to large ROI in four countries. These countries were undertaking substantial financing reforms and had demonstrated commitment to the work that SFI was undertaking. The political will demonstrated by these countries ensured the activities were successful. Understanding the right time and context for supporting these reforms will lead to the strongest chance of success. SFI demonstrated that trying to implement PFM reforms where there isn’t government commitment or financial protection activities where there isn’t already a health insurance scheme would not be a good use of resources.

Replicability of SFI successes would be dependent on the political and economic context of the country and less on the geographic location or disease area. SFI worked in 16 countries and 2 regional programs, and the biggest ROIs were in three East African countries and one Asian country. The success was due less to geographic location and more to the fact that these countries were undertaking health reform efforts and SFI was able to provide support at this critical time. Additionally, many of the SFI successes could be replicated outside of HIV/AIDS. The PFM efforts in Kenya led to an overall increase in health budget, as well as HIV/AIDS budget, as the reforms focused on the financing systems. In Vietnam, the efforts were specifically focused on integration of stand-alone HIV clinics into the social health insurance system; however, this effort could be studied by other disease areas for its policy success in implementing systematic reforms.

Although positive ROI came from only four countries, *not all interventions have immediate ROI*,* but those interventions can still be important and lead to systemic changes important for a more sustainable HIV/AIDS response.* As SFI progressed it became clear that while demonstrating results and having a clear focus on metrics was crucial, many financing activities may not have an ROI in a 3–5-year timeframe. This was seen in policy engagement and for activities with large start-up costs where the shorter time frame did not allow for the costs to be recouped (see Table 1). There are other impacts of SFI that were not quantifiable, such as better clinical outcomes, or that haven’t yet been realized but could have a positive ROI in future years.

Another lesson of SFI is that *increasing domestic financing was predicated on positive economic growth*–many PEPFAR countries had experienced significant economic growth in recent years, which assumed that these countries could take on a larger share of the HIV response. However, COVID demonstrated how a crisis can upend assumptions and planning. Many of the countries with the largest numbers of PLHIV and largest contribution of domestic financing for the HIV response prior to COVID also experienced the largest contraction of GDP due to COVID [9, 10]. However, innovations under SFI were of benefit during the pandemic. Decentralized drug delivery models were scaled up during COVID as clinics sought to decant patients from crowded public facilities. Integration of PLHIV and HIV into financial protection schemes allowed patients to seek holistic care. PEPFAR platforms, such as supply chain and facilities, were used to deliver COVID care. Expectation of how much countries can contribute to health and HIV/AIDS need to be tempered during a time of debt and economic crises for many PEPFAR countries. Systems improvements are still valuable investments but there is a need for realistic expectations of domestic resources for HIV/AIDS.

Work under SFI showed that *efficiencies of spending and HIV services are critical*,* especially when increased domestic financing is not feasible in the short term*. For example, in Tanzania a study produced by SFI found that multi-month dispensing (MMD) and differentiated care models could reduce the total economic cost of ART services by US$258 million over a five-year period [7]. In Namibia an efficiency study found that 52% of hospitals had technical inefficiencies, and if addressed, these facilities could save 32% in clinical staff costs and 46% in recurring purchases [7]. Considering shrinking fiscal space and limited domestic and donor resources, these types of efficiency gains are critical. Additionally, there needs to be more attention given to *linking tax reform efforts with work on increasing domestic resources for health.* Literature shows the importance of tax reform for increasing domestic resources for health; however it is difficult to fund tax reform efforts with earmarked health funds for HIV [2, 3]. Consideration should be given to how efforts in the economic growth space and health space can work together.

Similarly, *these types of financing interventions take substantial time and funding to see changes and there needs to be allowances for multi-year interventions*. At the time of SFI, PEPFAR used one-year budgeting and implementation cycles. This timing drives results and emphasizes the urgency needed to save lives. However, SFI was successful in achieving financing reforms because there was a longer timeline allowed for implementation. There would have been far fewer results if the financing activities had a one-year timeline. For example, it took about a year and half for the Nigeria private pharmacy drug distribution model to be designed and functioning during which there were no quantitative results. However, after three years of implementation, the model had an ROI of 45% and tens of thousands of clients had chosen to access their ARVs using this system.

While SFI demonstrated that dedicated attention and funding to health financing could yield results in budget increases, efficiencies, and increasing private sector engagement in service delivery, *clear budget communication from PEPFAR*,* as well as diplomatic engagement is needed to substantially increase country funding of the HIV/AIDS response.* SFI showed the impact of reforms in financial protection and budgeting which did have increases in domestic funding. However, as PEPFAR looks towards sustainability, the process for substantially increasing domestic funding for HIV will need to occur over a long-time horizon and will have to be a deliberative and step-wise approach. While there is no expectation that funding will substantially decrease in the next few years, it is still important to begin the dialogue using data that is increasingly available and chart a path towards a domestically funded HIV response.

## Conclusion

SFI demonstrated that by implementing dedicated and innovative approaches to HIV financing, PEPFAR funding can leverage additional resources from host-country governments and the private sector for a more sustainable HIV response. These financing interventions showed that a return on investment is possible and that they can lead to better clinical outcomes and increased client satisfaction. These interventions have the highest potential to succeed when they are designed with the country context in mind and with dedicated multi-year funding. As PEPFAR looks towards the future and focuses on sustainability and country ownership of the HIV/AIDS response, the lessons learned from SFI will be critical in designing country programs. Further attention should be given to scaling up the successful financing models deployed under SFI, and consideration should be given to funding these interventions with central funding until they are priorities at a country level.

## Data Availability

The data used are available on reasonable request.

## Abbreviations

ART: Antiretroviral therapy
ARV: Antiretroviral drugs
CDU: Central dispensing unit
CRDB: Cooperative Rural Development Bank
CSO: Civil society organization
DCA: Development credit authority
DRM: Domestic resource mobilization
HIVST: HIV self testing
MMD: Multi-month dispensing
NGO: Non-governmental organization
PEPFAR: President’s Emergency Plan for AIDS Relief
PFM: Public financial management
PLHIV: People living with HIV/AIDS
ROI: Return on investment
SFI: Sustainable Financing Initiative
SHI: Social health insurance
UHC: Universal health coverage
USAID: U.S. Agency for International Development
USG: United States government
WHO: World Health Organization
